# Supplementary material for: Complex epistatic interactions between ELF3, PRR9, and PRR7 regulate the circadian clock and plant physiology
Source: Genetics. 2023 Dec 24;226(3):iyad217. doi: 10.1093/genetics/iyad217 (PMC10917503; doi:10.1093/genetics/iyad217)
Supplement: iyad217_Supplementary_Data [file iyad217_supplementary_data.zip › Supplementary_Figures_1_-_6_GENETICS-2023-306643.pdf]

# Supplementary Figure 1

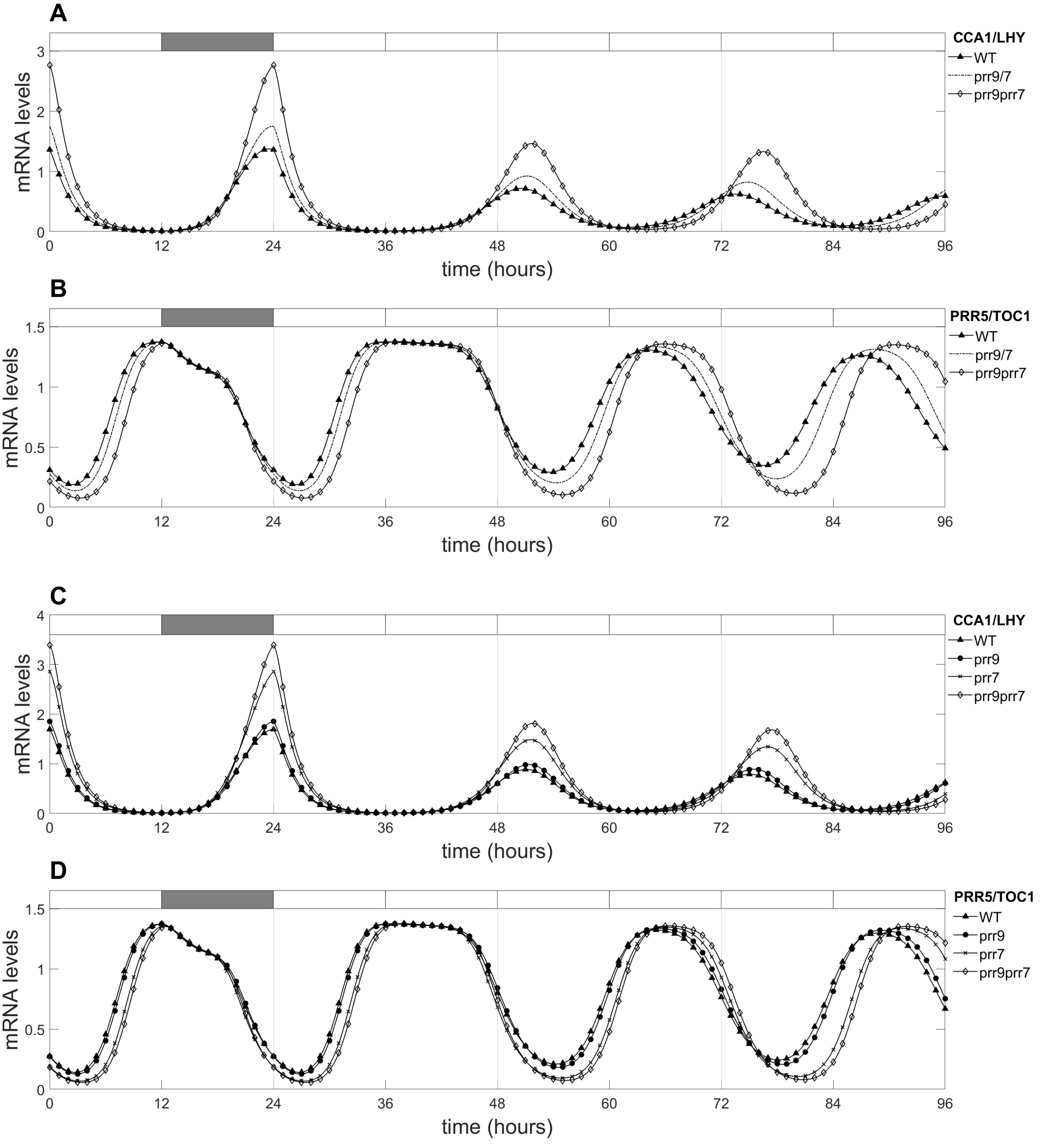


<u>Supplementary Figure 1 – Simulating mutations in model 1 and model 2 replicates experimental data.</u> To determine whether the modifications in (A) model 1 and (B) model 2 could replicate known circadian phenotypes, we simulated the *prr9*, *prr7* and *prr9/prr7* mutations by setting the respective gene's transcription rate to zero. For model 1, the outputs for the *prr9* and *prr7* are combined into a single output (*prr9/7*) as model 1 keeps the same function for the respective single mutants.

# Supplementary Figure 2

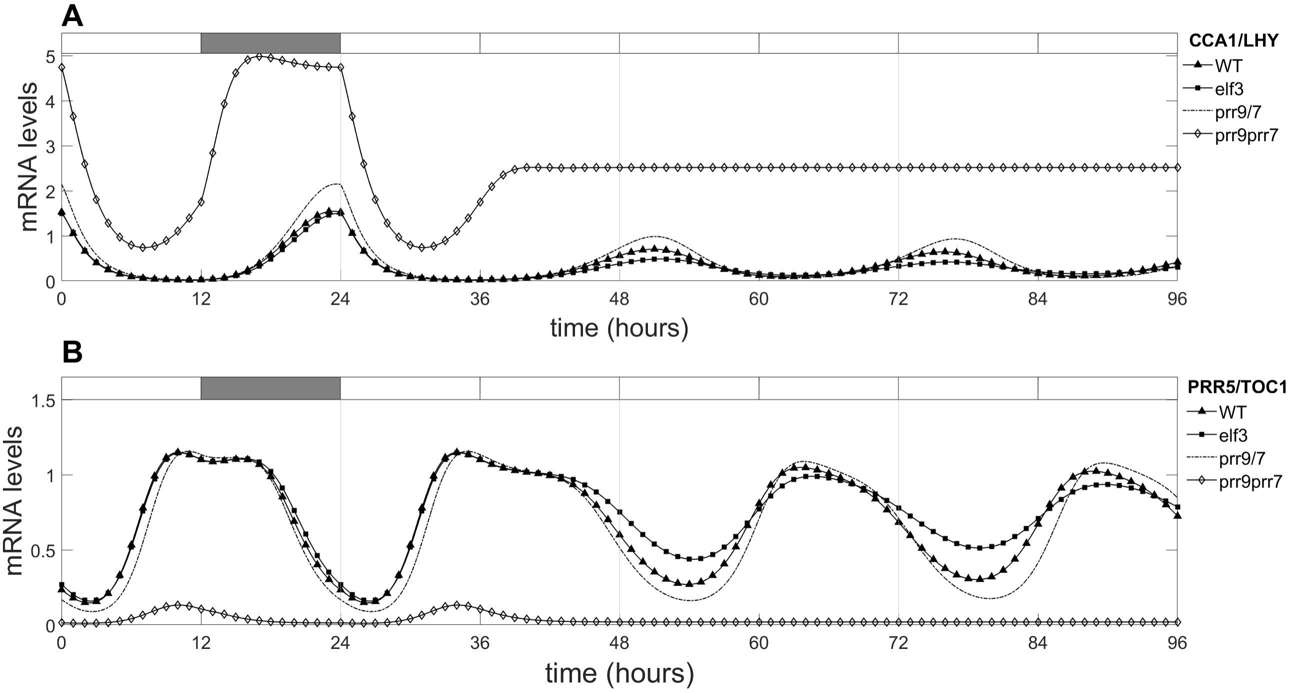


Supplementary Figure 2 – Model 3 does not replicate experimental data. In the third modification of the DC2016 model, we split the P97 component of the adaptation in Greenwood *et al.*, 2022 into two separate components termed P9 and P7. The outputs of (A) *CL* and (B) *P51* in the WT and simulated *elf3/prr* mutant backgrounds are shown.

# Supplementary Figure 3

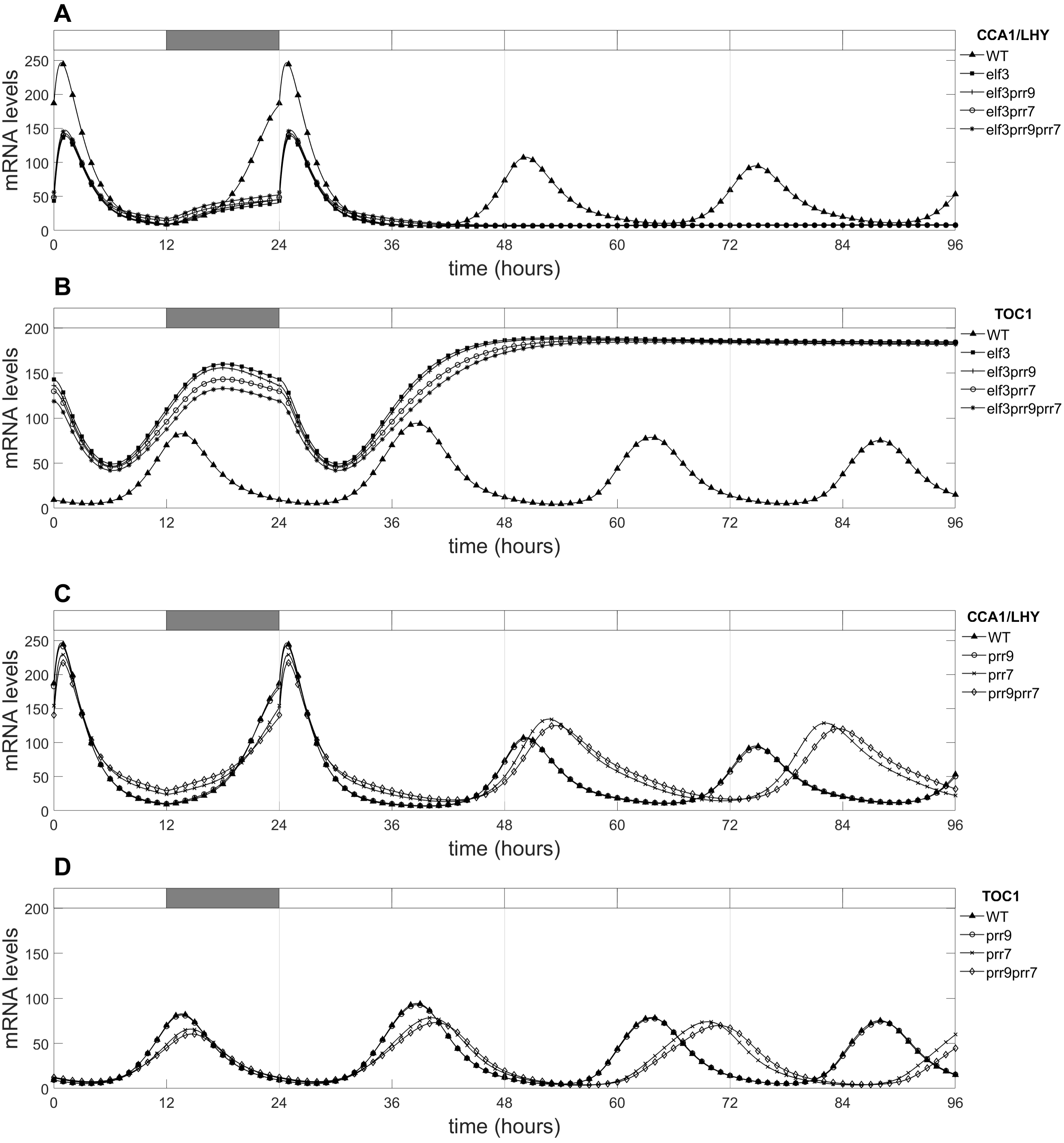


<u>Supplementary Figure 3 – The outputs from the *elf3/prr9/prr7* mutant are arrhythmic in the Urquiza-García 2019 model.</u> Using the U2019 model, we simulated the expression of either (A-C) *CCA1/LHY* or (B-D) *TOC1* in a wild-type, single, double, and triple *elf3/prr9/prr7* context. The mutants were implemented as described in the materials and methods.

# Supplementary Figure 4

**A**

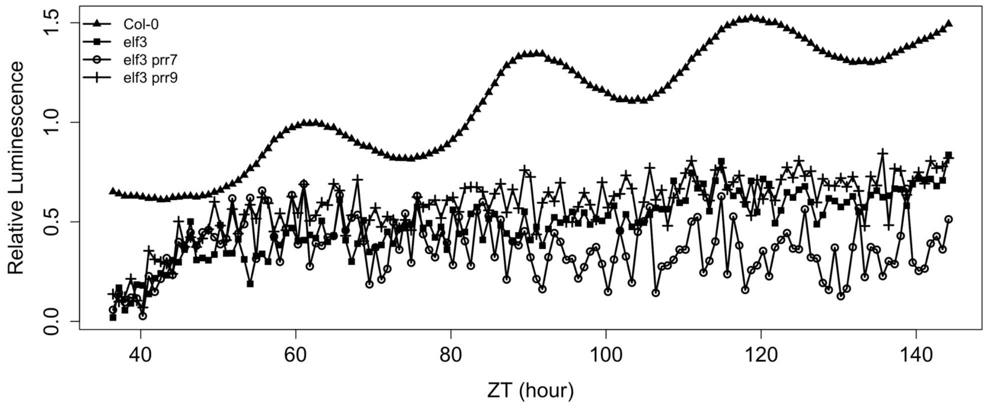


**B**

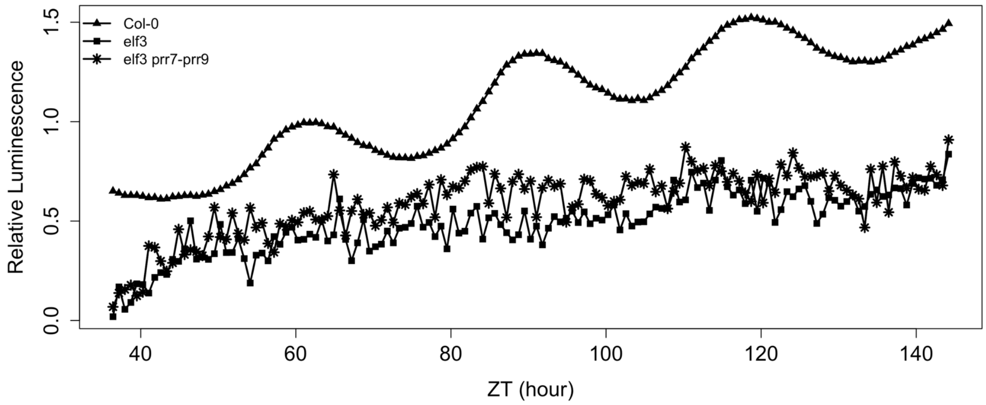


<u>Supplementary Figure 4 – The *prr9* or *prr7* mutations does not rescue the arrhythmicity of the *CCA1::LUC* reporter in the *elf3* background</u> The arrhythmicity of the *CCA1::LUC* reporter in the *elf3-1* background is not restored by the (A) *prr9* or *prr7* single mutations. (B) The arrhythmicity of the *elf3 CCA1::LUC* was also not restored by simultaneous mutations in *prr9 and prr7*. Seedlings were entrained under neutral-day (12/12) cycles before being released into constant light and temperature. The first day under constant conditions is not shown.

# Supplementary Figure 5

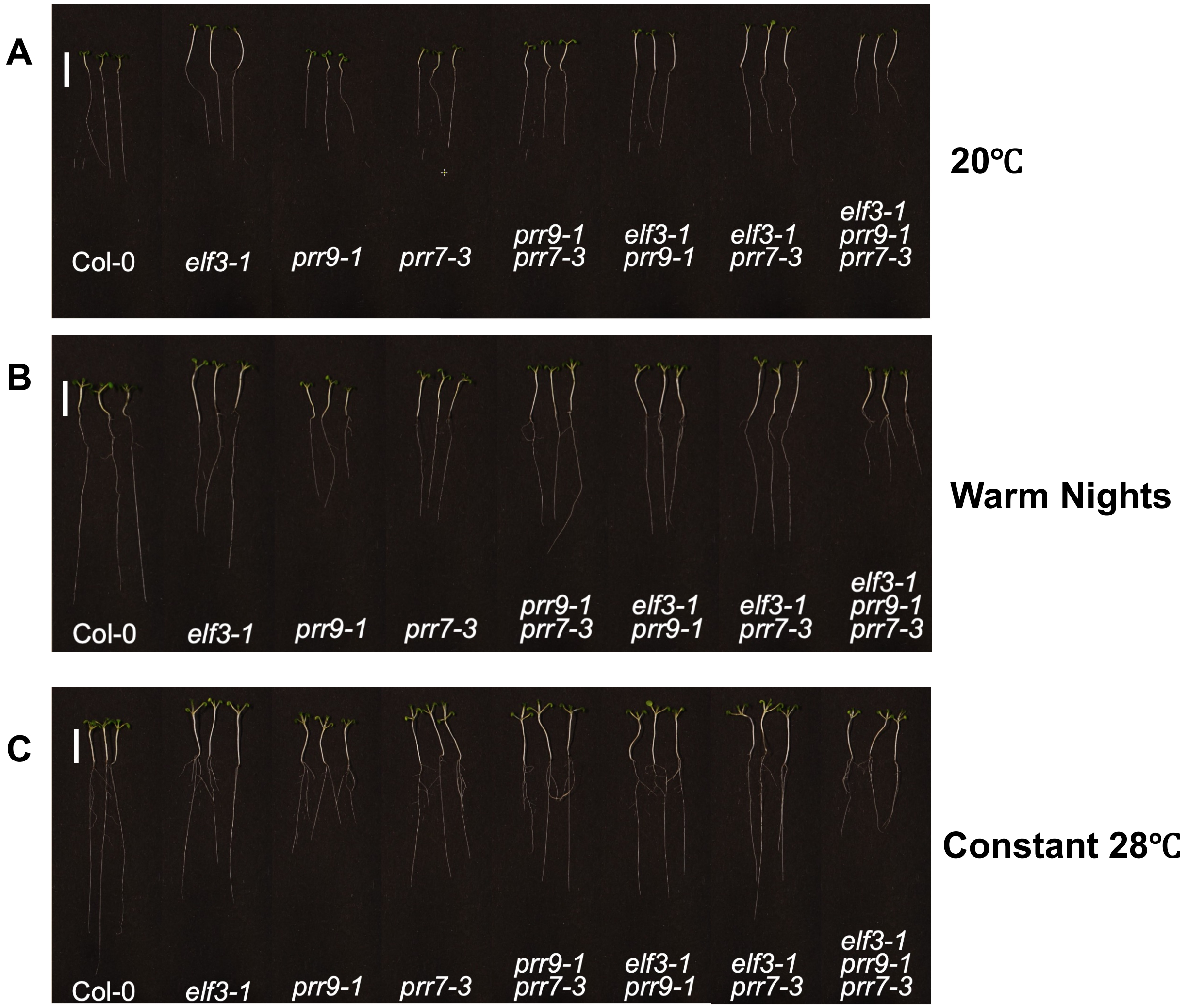


Supplementary Figure 5 – Representative images of the *elf3* and *prr* lines. Images of seedlings grown under short-day photoperiods with (A) 20°C, (B) warm 28°C nights only and (C) constant warm (28°C) temperatures. Scale bars are 10 mm.

# Supplementary Figure 6

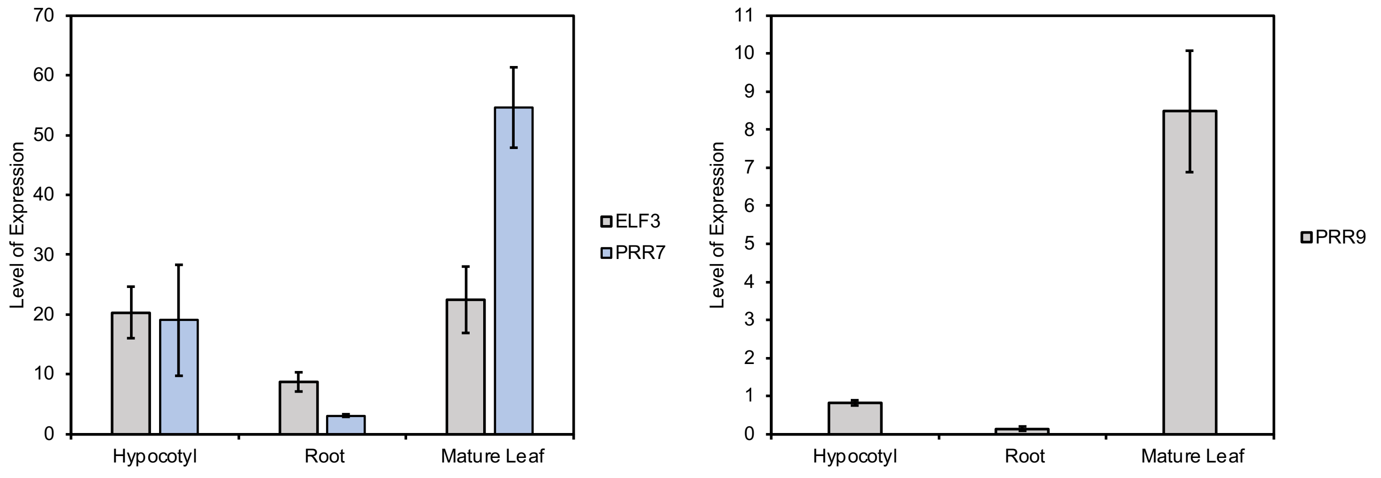


Supplementary Figure 6 – Expression profile of *ELF3, PRR9 and PRR7* in different tissue types. The absolute expression of *ELF3*, *PRR9* and *PRR7* in the seedling hypocotyl, seedling root and mature leaf. The data is from Klepikova *et al.,* (2016) and were accessed from the Arabidopsis eFP browser (Winters et al., 2007 and Sullivan et al., 2019). Error bars are standard deviation.
