# Supplementary material for: Complex epistatic interactions between ELF3, PRR9, and PRR7 regulate the circadian clock and plant physiology
Source: Genetics. 2023 Dec 24;226(3):iyad217. doi: 10.1093/genetics/iyad217 (PMC10917503; doi:10.1093/genetics/iyad217)
Supplement: iyad217_Supplementary_Data [file iyad217_supplementary_data.zip › Supplementary_Table_1_GENETICS-2023-306643.pdf]

Supplementary table 1 – qPCR primers used in this work.

**Name**	**Sequence**
CCA1-RT-F	CCAGATAAGAAGTCACGCTCAGAA
CCA1-RT-R	GTCTAGCGCTTGACCCATAGCT
LHY-RT-F	GACTCAAACACTGCCCAGAAGA
LHY-RT-R	CGTCACTCCCTGAAGGTGTATTT
IPP2-RT-F	GTATGAGTTGCTTCTCCAGCAAAG
IPP2-RT-R	GAGGATGGCTGCAACAAGTGT
